# Aurora Kinase A Regulation by Cysteine Oxidative Modification

**DOI:** 10.3390/antiox12020531

**Published:** 2023-02-20

**Authors:** In-Gyun Lee, Bong-Jin Lee

**Affiliations:** 1 Biomedical Research Division, Korea Institute of Science and Technology, Seoul 02792, Republic of Korea; 2 Research Institute of Pharmaceutical Sciences, College of Pharmacy, Seoul National University, Seoul 08826, Republic of Korea

**Keywords:** Aurora kinase A (AURKA), redox-active molecules, cysteine oxidative modification

## Abstract

Aurora kinase A (AURKA), which is a member of serine/threonine kinase family, plays a critical role in regulating mitosis. AURKA has drawn much attention as its dysregulation is critically associated with various cancers, leading to the development of AURKA inhibitors, a new class of anticancer drugs. As the spatiotemporal activity of AURKA critically depends on diverse intra- and inter-molecular factors, including its interaction with various protein cofactors and post-translational modifications, each of these pathways should be exploited for the development of a novel class of AURKA inhibitors other than ATP-competitive inhibitors. Several lines of evidence have recently shown that redox-active molecules can modify the cysteine residues located on the kinase domain of AURKA, thereby regulating its activity. In this review, we present the current understanding of how oxidative modifications of cysteine residues of AURKA, induced by redox-active molecules, structurally and functionally regulate AURKA and discuss their implications in the discovery of novel AURKA inhibitors.

## 1. Introduction

Aurora kinase A (AURKA), a serine/threonine kinase family member, plays a fundamental role in various aspects of cellular processes [1,2], such as regulating centrosome maturation and spindle formation during mitosis, entry into the mitotic phase, and mitotic division [2]. Recently, several non-mitotic roles of AURKA have been reported, further implicating the role of AURKA in several important cellular processes [3,4,5]. Since its discovery in the mid-1990s, AURKA has drawn much attention; AUKRA protein overexpression, gene amplification, and its mutations were reported to be associated with carcinogenesis, leading to poor prognosis. Therefore, AURKA is considered as an appealing target for the therapeutic interventions [2,6,7,8,9,10,11,12]. Several strategies of targeted and specific inhibition of AURKA led to the development of a new class of drugs known as AURKA inhibitors [13,14]. To date, several AURKA inhibitors have successfully reached to the clinical trials for the treatment of diverse cancers including leukemia, triple-negative breast cancer, and prostate cancer [15,16]; Most of the clinically effective AURKA inhibitors were ATP-competitive inhibitors, with IC_50_ values lying typically in the sub-nanomolar range [15,16]. Despite their high-potent inhibition against the enzymatic activity of AURKA, AURKA inhibitors typically have severe on-target as well as off-target toxicities, such as bone marrow and epithelial cells, leading to severe adverse effects such as neutropenia, mucositis, and somnolence, severely limiting the therapeutic window of the AURKA [17,18,19,20] inhibitors.

To overcome these issues and develop more selective AURKA inhibitors, many researchers focused on developing a new class of AURKA inhibitors targeting diverse regulatory factors that affect AURKA function. Through decades of intensive research, other factors, including co-factors interacting with AURKA, AURKA-substrates, and post-translational modifications such as cysteine oxidation, have been identified to be crucial for the activity and specific localization of AURKA [21,22,23,24,25,26,27,28,29,30,31,32]. These findings provided a rationale for developing new therapeutic strategies involving the control of AURKA activation pathways, to completely block the enzymatic and non-enzymatic function of AURKA and overcome on-target/off-target toxicities.

For instance, several small molecules that modulate the interaction between AURKA and its major cofactor, targeting protein for Xklp2 (TPX2), have been developed [33,34,35,36]. TPX2 uses its N-terminal 43 residues to form a tight complex with AURKA at the spindle microtubule, where it activates the AURKA by (i) inducing the structural alteration of AURKA into catalytically competent structure and (ii) inhibiting the dephosphorylation by phosphatases [21,34,37,38,39,40]. Several small-molecule inhibitors demonstrated their inhibitory effect against enzymatic activity of AURKA, by specifically blocking the TPX2-AURKA interactions rather than ATP binding sites [33,34,35,36].

Interaction of AURKA with N-MYC oncoprotein, another example of a cytosolic AURKA-binding protein [41], can be exploited to develop therapeutic interventions. MYC functions as a transcription factor that orchestrates the downstream oncogenic signaling networks, which are frequently activated in multiple human cancers, such as breast cancer, liver cancer, colorectal carcinoma, and prostatic neoplasia [42,43]. Hence, the MYC protein can be considered as an ideal target for the cancer treatment. However, lack of a preferred binding pocket for traditional drug modalities poses a demand for alternative strategies to indirectly inhibit MYC function, one of which involves exploiting the interaction between MYC and AURKA [41,44,45]. N-MYC protein is stabilized through the complex formation with AURKA that protects N-MYC from proteasomal degradation [45]. Several small-molecule inhibitors have successfully disrupted the interaction between N-MYC and AURKA, leading to the destabilization and degradation of N-MYC and subsequently tumor regression in *MYCN* amplified cancers [46,47,48].

In addition to the aforementioned intermolecular protein-protein interactions regulating AURKA activity, several evidences have demonstrated that the activity of AURKA depends on the redox modifications of the cysteine residues in its kinase domain. Although therapeutic significance of the redox modifications of AUKRA is rarely reported [49], a greater understanding of redox-based AURKA regulation may open new therapeutic avenues for developing effective AURKA inhibitors with novel mechanisms of action. Here, we discuss our current understanding of the multifaceted effects of redox-active molecules on the activity of AURKA.

## 2. Redox-Based Cell Cycle Regulation

Diverse cellular metabolic activities in various organelles such as mitochondria, peroxisomes, and phagosomes, result in the production of reactive oxygen species (ROS), a collective term for all highly reactive oxygen derivatives produced by the partial reduction of molecular oxygen [50,51,52]. Although ROS have long been considered as toxic byproducts affecting normal cell function by inducing unwanted oxidative stress, it is accepted that ROS at physiological levels plays a critical role in signaling and are essential for maintaining overall cellular homeostasis [53,54,55]. In particular, the redox state of cells at each divisional state is critical for the normal cell proliferation. During the cell cycle of proliferating eukaryotic cells, the intracellular pH and balance between redox pairs such as NADH/NAD+ and NADPH/NADP+ continuously oscillate; hence, proper cellular machinery is required to sense and respond to these alterations [56]. A body of evidences indicates that ROS regulates and controls the cell cycle (e.g., G2/M transition) by specifically modulating the redox-sensitive protein associated with cell cycle regulation [57,58]. For instance, ROS can directly regulate the phosphatase cell division cycle 25 (CDC25), which is essential for controlling cell-cycle progression [59], including the G2/M transition through dephosphorylation of the inhibitory phosphorylation sites of cyclin-dependent kinase (CDK)/cyclin complexes [60]. This event activates the CDK/cyclin complex, facilitating the progression through cell division [61]. ROS, such as H_2_O_2_, induces the formation of intracellular disulfide bonds between the highly reactive cysteine residues Cys330 and Cys377 of CDC25, leading to the inactivation of the CDC25, by which the activation of the CDK/cyclin complex is induced [62].

ROS-mediated regulation of protein phosphatase 2A (PP2A) is also involved in redox-mediated cell cycle regulation. It belongs to the phosphoprotein phosphatases (PPP) family, which has been detected to control a myriad of protein dephosphorylation events in cells [63], controlling key cellular processes such as signal transduction, protein translation, immune regulation and most importantly, mitosis [64]. PP2A functions antagonistically to mitotic kinases, such as AURKA and CDK1, by counteracting mitotic kinase-induced phosphorylations in most eukaryotes [65,66]. Furthermore, PP2A negatively regulates CDC25 [66]. By dephosphorylating Thr130 residue in CDC25, PP2A promotes the formation of the 14-3-3 and CDC25 complex, and subsequent cytosolic sequestration of CDC25 [67]. The regulatory dephosphorylation activity of CDC25 critically depends on its interaction with various ROS species, such as H_2_O_2_, nitric oxide, and peroxynitrite, which pose specific functional impacts on PP2A [68,69,70,71,72]. In solution, PP2A exists primarily as a heterotrimer consisting of a catalytic subunit (PP2Ac) complexed with a scaffold and regulatory subunits [73]. Each subunit has multiple isoforms; therefore, variable combinations of the three subunits forming the holoenzyme can influence the subcellular localization and substrate specificity. The catalytic subunit PP2Ac contains 10 cysteine residues, including a canonical CXXC motif at position 266–269, that can undergo reversible intra- and inter- molecular disulfide bonds formation [71,72]. The formation of disulfide bonds induces the structural alterations leading to the inhibition of PP2Ac activity, allowing PP2A to sense oxidative stress and subsequently regulate the cell cycle by modulating downstream signaling pathways [71,72].

The aforementioned redox-sensitive proteins contain highly reactive cysteine residues, which allows them to translate oxidative changes into structural rearrangements and functional consequences of the proteins. Similarly, AURKA contains several reactive cysteine residues that can undergo redox modifications, as discussed in detail below, enabling AURKA to sense and respond to the oscillating cytoplasmic redox states during the cell cycle.

## 3. Cysteine Residues in AURKA

The sulfur-containing cysteine residue is capable of undergoing diverse oxidative modifications in response to a wide variety of oxidative stresses. This feature of cysteine supports a wide range of organisms to cope with environmental stresses [74]. Among methionine and cysteine, the two sulfur-containing amino acids present in the proteins, the thiol functional group of cysteine can undergo a wide variety of modifications, such as oxidation to sulfenic (R-SOH), sulfinic (R-SO_2_H), and sulfonic (R-SO_3_H) acids, as well as the formation of disulfide bonds (R-S-S-H), which allow cysteine-containing proteins to regulate a wide variety of biological processes in a very exquisite way [75]. However, only certain cysteine residues in the protein can undergo such modifications; this fact emphasizes the significance of the microenvironment surrounding the cysteine residue, which affects the reactivity [76]. The susceptibility of cysteine to these redox modifications is largely dependent on the reactivity of each specific sulfhydryl group, strongly influenced by solvent accessibility, the polarity of surrounding residues, and the pH [77,78]. The ionization constants (pKa) for the equilibrium between free cysteine thiol (-SH) and thiolate (-S-) is approximately 8.5, similar to the cytoplasmic pH [78,79,80,81]. However, the pKa of cysteine residues of a protein or the peptide can vary drastically. For example, the electrostatic field associated with an α-helix pointing with its N-terminus towards the cysteine residue has been shown to lower the thiol pKa value by up to ~5 in several proteins [82]. Therefore, taking into account structural information is imperative when predicting the structural and regulatory functions of cysteine residues against oxidative stress [83,84].

**Figure 1:**
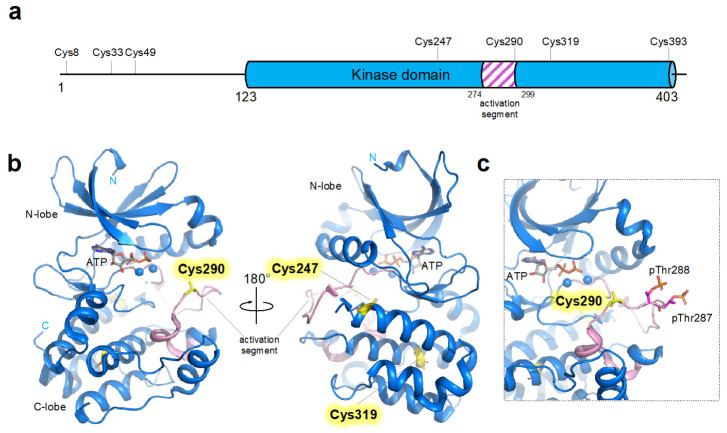
Domain organization and overall structure of human AURKA. (**a**) Domain organization of N-terminal auxiliary domain (residues 1–122) and kinase domain (residues 123–403) comprising the human AURKA. Cysteine residues and activation segments (residues 274–299) are also marked. (**b**) Crystal structure (shown as ribbon representation) of AURKA bound to ATP (PDB ID: 1OL5). Cysteine residues located in the c-lobe are shown as yellow sticks, while the cys393 located at the disordered c-terminal residue of AURKA could not be shown. (**c**) Close-up view of the active site (ATP binding pocket) of AURKA. The phosphorylated threonine residues (Thr287 and Thr288) are shown as sticks, and the activation segment is colored as pink.

AURKA consists of a non-conserved, flexible auxiliary N-terminal domain and a C-terminal conserved kinase domain (Figure 1). AURKA contains three cysteine residues (at amino acids positions 8, 33, and 49) in the N-terminal auxiliary domain and four cysteine residues (at amino acids positions 247, 290, 319, and 393) in the C-terminal kinase domain (Figure 1a). Unlike the kinase domain, the N-terminal domain of AURKA still needs to be further studied to clarify its function. However, several studies have explored the functional and structural role of the N-terminal domain, including a docking site for AURKA cofactors, or implications for autoinhibitory interactions [85,86]. As the function of cysteine residues in the N-terminal domain has not been systemically tested, we focused on the role of cysteine residues in the kinase domain of AURKA. Similar to other kinases, AURKA displays a canonical bilobal fold consisting of N-, and C-lobes and an ATP-binding cleft between the lobes (Figure 1b) [87]. All four cysteine residues in the kinase domain of AURKA are located in the C-terminal lobe of the kinase, and only Cys290 and Cys393 are exposed to the solvent surface [87]. Previous reports detected that mutation of these solvent exposed residues improves the stability of the protein and forces the protein to exist as a monomer in solution, suggesting that those solvent-exposed cysteine residues are involved in the formation of inter-molecular disulfide bonds leading to the formation of homodimer in higher multimers [88]. Nevertheless, further investigation is needed to confirm the physiological roles of the formation of inter-molecular disulfides. Of the two surface-exposed residues, Cys290 seems to play a critical role, as it lies at the center of the activation segment of the kinase domain, while Cys393 is located at the C-terminal flexible tail (Figure 1c). The kinase activity of the AURKA depends on and is regulated by the phosphorylation state of the strictly conserved residue, Thr288, located within the activation segment, a conserved structural element in most eukaryotic kinase families. It is now well established that the conformation of the activation segment consisting of Asp-Phe-Gly (DFG) motif, activation loop which contains the site of regulatory phosphorylation (Thr288 in AURKA), and APE (Ala-Pro-Glu) motif, controls kinase activity [89] The phosphorylation of Thr288 leads to the global structural reorganization required for the activation of AURKA, involving remodeling and releasing the autoinhibited “DFG-in” state and conformational change of the activation segment that enables the binding of substrate [89]. As the conformation of the activation segment critically determines the kinase activity, the redox modification of Cys290, which is located in the vicinity of Thr288, is expected to significantly affect the overall activity of the kinase.

Although Cys247 and Cys319 are buried within the interior of the protein compared to the location of Cys393 and Cys290, a crystallographic study has shown that the Cys247 can also be covalently modified [30]. When AURKA was crystallized in the presence of sodium cacodylate, an organic arsenic compound commonly used as a buffering agent during crystallization process, Cys247 was found to be covalently modified with dimethyl arsenic adducts [30]. A further study of the physiological functions of Cys247 modification would suggest the possible role of Cys247 modification in regulating AURKA activity.

## 4. Functional and Structural Consequences of Cysteine Modification in AURKA

### 4.1. Two Distinct Pathways of AURKA Activation

Spatiotemporal regulation of AURKA’s activity is multifactorial. A growing body of evidences suggests the following two main pathways that active AURKA: (i) the phosphorylation of conserved Thr288 residing on the activation segment or the (ii) interaction with co-factor proteins (e.g., TPX2) that induces the structural rearrangement competent for the phosphotransfer activity [37,38,39,88,90,91,92,93,94,95]. Although the phosphorylation of Thr288 and TPX2 binding synergistically activate AURKA in vitro [91,96], two distinct pathways seem to work independently in an intracellular environment [23,97,98,99]. As discussed in detail below, biochemical, biophysical, structural, and cellular evidences have shown that the redox modification of the cysteine residues of AURKA affects both the phosphorylation state of Thr288 and the conformation state of structural elements critical for the kinase activity, leading to the regulation of AURKA activity.

### 4.2. H_2_O_2_ Induced Oxidative Modification of Cysteine

Hydrogen peroxide (H_2_O_2_) is a major ROS member that can act as a signaling molecule in many biological systems [100]. Endogenously, H_2_O_2_ can be produced as a result of [32] metabolic reactions such as respiration, and is implicated in many redox signaling pathways [101,102]. Furthermore, H_2_O_2_ levels have also been implicated in the regulation of mitosis by directly oxidizing various kinases and phosphatases involved in the cell cycle regulation [103,104,105,106]. Considering the variable intracellular H_2_O_2_ level in multiple stages of the cell cycle [107,108], cells can be considered to have critical ways of sensing intracellular H_2_O_2_ levels and responding to them. For example, high levels of H_2_O_2_ have been shown to induce the cell cycle arrest, and relatively low levels of H_2_O_2_ are required for mitotic entry [32,107,109].

So far, several lines of evidence have shown that H_2_O_2_-induced regulation of the AURKA activity, possibly through direct oxidation of Cys290 present in the activation segment of the kinase domain. In mammalian cells, the oxidative stress induced by the additional H_2_O_2_ results in the mitotic delay and abnormal mitotic spindle formation [32]. As the spindle formation is the process that is mainly governed by the kinase activity of AURKA, these observations led to the hypothesis that H_2_O_2_ regulates the activity of AURKA [110,111]. Indeed, the phosphorylation level of Thr288 of AURKA was significantly elevated when the mammalian cells were treated with H_2_O_2_, suggesting that the hyperphosphorylation of AURKA induces mitotic delay and abnormal spindle formation [32].

In contrast, when the purified recombinant AURKA was treated with H_2_O_2_, its overall kinase activity of AURKA (as measured in terms of the phosphorylation level of fluorescent-tagged substrate peptide) was decreased, while the phosphorylation level of Thr288 remained almost unchanged [29]. The mutational analysis further confirmed that the Cys290 was the main site for H_2_O_2_-induced oxidative modifications, and the inhibitory effect of H_2_O_2_ on AUKRA activity was reversed by almost equimolar concentrations of the reducing agent dithiothreitol (DTT), suggesting a reversible inhibitory effect of H_2_O_2_ on AURKA activity [29]. Interestingly, adding relatively higher amounts of DTT (~100 mM) to the *Xenopus laevis* (*X. laevis*) egg extract system, a powerful tool for studying the cell cycle at the molecular level, inhibited the phosphorylation of Thr295 in the *X. laevis* AURKA (equivalent to Thr288 of human AURKA) [30]. These observations suggest the presence of indirect or cell cycle-specific signaling pathways that lead to elevated Thr288 phosphorylation levels and AURKA activation. As the phosphorylation state of Thr288 alone does not fully reflect the enzymatic activity of the kinase, direct measurements of kinase activity of AURKA in addition to Thr288 phosphorylation level at the specific intracellular localization and timing in cells would aid confirming the interplay of diverse AURKA cofactors that regulates the activity.

### 4.3. Structural Transitions Induced upon Covalent Modification of Cysteine in AURKA with Coenzyme A

Coenzyme A (CoA) is a fundamental metabolic cofactor that participates in numerous biological metabolic processes [112]. It particularly plays a central role and functions as an obligate cofactor in energy and fatty acid metabolic pathways [113]. As CoA contains a thiol group, it can interact with other cellular thiols to form a disulfide bond and can also covalently modify protein thiols in cysteine or methionine amino acids [112]. Covalent modification with CoA (CoAlation) of cysteine residues plays a role in post-translational modification, which can lead to altered enzyme activity, protein-protein interactions, and localization [112,114,115]. Interestingly, it has been reported that CoA and its derivatives regulate the activity of several protein kinases, such as PKC (protein kinase C), CaMKII, and AURKA through direct activation or inhibition [116,117]. Several CoAlated structures of AURKA have enabled a deeper understanding of CoA-mediated regulation of AURKA activity at the structural and molecular levels.

**Figure 2:**
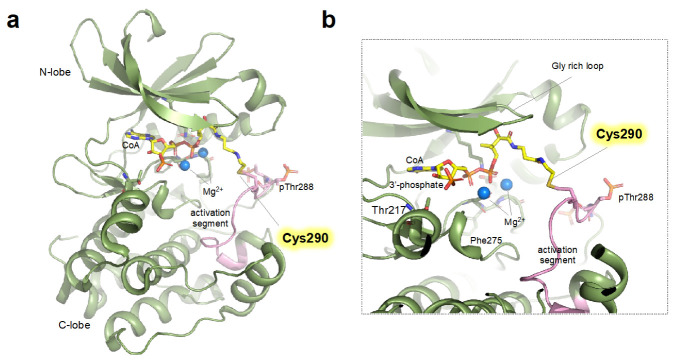
Structure of monomeric CoAlated AURKA structure (**a**) Crystal structure (shown as ribbon representation) of AURKA bound to CoA (PDB ID: 6I2U) displaying monomeric structure (See main text for details). (**b**) Close-up view of the active site (ATP binding pocket) of CoAlated AURKA. Cys290 forming covalent bonds with CoA is shown as yellow stick and activation segment is colored in pink.

The previous study reported by Tsuchiya et al. revealed the detailed biophysical and structural basis of the CoA-mediated AURKA inhibition [31]. Kinome-wide screening of CoA against various protein kinases revealed that CoA specifically inhibits the catalytic activity of AURKA. Mass spectrometric and mutational analyses confirmed that the CoA molecule covalently modified the Cys290, and the modification of Cys290 decreased AURKA phosphotransferase activity towards myelin basic protein substrate in a dose dependent manner [31]. The crystal structure of CoAlated Cys290 AURKA elucidated the structural basis of the inhibitory effect of Cys290 CoAlation (Figure 2). In the crystal structure, the 3′-phospho-ADP moiety of CoA was bound to the ATP-binding pocket of the kinase domain of AURKA, suggesting that the CoA can occupy the ATP-binding pocket and thus compete with cellular ATP (Figure 2b). The pantetheine moiety of CoA stretches toward the catalytic activation segment of AURKA, allowing the sulfhydryl group to form a disulfide bond with the Cys290 located in the vicinity of Thr288.

The structural prerequisite for sufficient activation of AURKA is the presence of several structural elements such as “DFG-in”, “α-Helix in”, and the salt bridge between catalytically important Lys162 and Glu181 [90]; interestingly, the CoA-bound AURKA harbors these hallmarks of the active conformation. However, covalent modification of the Cys290 resides in the “P + 1 loop”, a portion of the activation segment that constitutes a binding site for substrate peptide, would sterically constrain the P + 1 loop, leading to impaired geometry unsuited for substrate binding (Figure 2b) [89,118]. Furthermore, the covalent binding of CoA resulted in the loss of hydrogen bonding between Arg255 (the central residue of the catalytic HRD motif) and phosphorylated Thr288, which would further impair the ideal geometry for the catalysis [119]. Interestingly, the binding of TPX2, a major cofactor of the AURKA, almost completely blocks the inhibitory effect of CoA [31]. This indicates that, in the presence of TPX2 (i.e., spindle pool of AURKA), the inhibitory effects of CoA are expected to be significantly limited. Whether the other major cofactors (e.g., CEP192 or Bora) also block the inhibitory effect of CoAlation of cysteine should be further determined.

**Figure 3:**
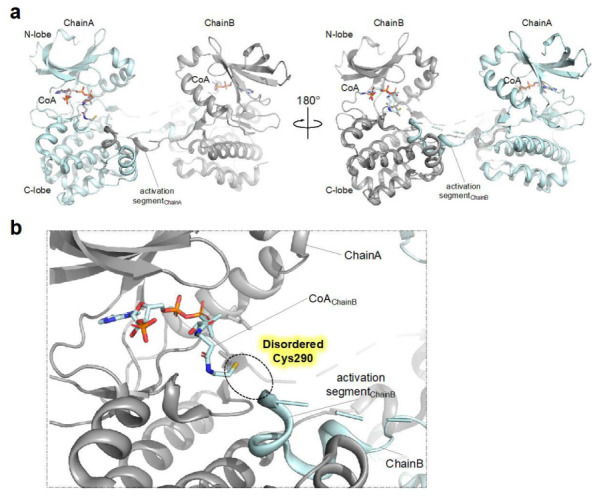
Structure of dimeric CoAlated AURKA structure (**a**) Crystal structure (shown as ribbon representation) of AURKA bound to CoA (PDB ID: 6XKA) displaying dimeric structure. (**b**) Close-up view of the active site (ATP binding pocket) of CoAlated AURKA. As the structure contains only one molecule in the asymmetric unit, Chain B denotes the AURKA molecule present in the neighboring asymmetric unit, not in the same unit. Also, the portion of the activation segment containing Cys290 is disordered and therefore not modeled in the structure.

Although the study by Tsuchiya et al. detailed the structural and biophysical basis for CoA- mediated AURKA inhibition, the effect of CoAlation on AURKA does not seem straightforward, as other Cys290 CoAlated structures have shown the structural implication of CoAlation for the AURKA activation (Figure 3) [30]. A study by Lim et al. reported a CoA-bound structure displaying an activation segment-swapped homodimer, a more catalytically competent conformation (Figure 3). Contrary to the monomer structure reported by Tsuchiya et al. this structure contained a homodimer and CoA molecules covalently bounded to the Cys290 residues of neighboring monomers, suggesting that the formation of the Cys290-CoA covalent bond may facilitate stabilization of the activation segment swapped dimeric structure, which would consequently promote the autophosphorylation of Thr288 (Figure 3b). Whether the CoAlation leads to the formation of dimeric structure in solution needs further investigation. As the authors used a TPX2-fused chimeric protein, with the N-terminal AURKA residues (at positions 1–115) replaced with TPX2 (at positions 7–20), the structure might reflect that of the distinct CoA-bound AURKA in the presence of TPX2.

Due to the flexible nature of the kinase activation segment, several distinct structural populations can exist in solution, and relatively minor conformations that are prone to crystallize can be captured in crystal structure. Therefore, concluding solely from the crystal structure could often be misleading. Other biophysical and cellular experimental approaches reflecting more physiological conditions would complement the information obtained from the crystal structure analyses and aid the comprehensive understanding of CoA-mediated regulation of AURKA.

## 5. Conclusions

Given its critical role in cell division and cancer, there has been great interest in developing inhibitors targeting AURKA over the last two decades [13,16]. Although several potent ATP- competitive AURKA inhibitors have been successfully progressed into clinical trials, the identification of the regulatory mechanisms affecting the localization and activity of AURKA is crucial to overcome side effects and to block the non-catalytic role of AURKA [3,5,13,15,16,44,45,48]. Here, we reviewed several examples of redox-based mechanisms for AURKA regulation. Despite our limited understanding of the impact of redox-active molecules on the catalytic function of AURKA, redox-active molecules can directly influence the function and structure of AURKA. The structure of CoA-bound AURKA is particularly interesting; CoA molecules can block the ATP-binding site and compete with ATP; also, it structurally alters the activation segment by covalently modifying the Cys290 residing in the segment [30,31]. As the diverse covalent kinase inhibitors targeting cysteine in the vicinity of ATP-binding pocket have exhibited superior efficacy and selectivity [120], supporting its approval by the FDA, Cys290 of AURKA is suggested to be a new target for developing AURKA inhibitors.

## Data Availability

All data needed to evaluate the conclusions in the paper are available from the corresponding author upon request.

## References

[1] Carmena M., Earnshaw W.C. (2003). The cellular geography of aurora kinases. Nat. Rev. Mol. Cell Biol..

[2] Vader G., Lens S.M. (2008). The Aurora kinase family in cell division and cancer. Biochim. Biophys. Acta -Rev. Cancer.

[3] Adhikari B., Bozilovic J., Diebold M., Schwarz J.D., Hofstetter J., Schröder M., Wanior M., Narain A., Vogt M., Dudvarski Stankovic N. (2020). PROTAC-mediated degradation reveals a non-catalytic function of AURORA-A kinase. Nat. Chem. Biol..

[4] Mahankali M., Henkels K.M., Speranza F., Gomez-Cambronero J. (2015). A non-mitotic role for Aurora kinase A as a direct activator of cell migration upon interaction with PLD, FAK and Src. J. Cell Sci..

[5] Guarino Almeida E., Renaudin X., Venkitaraman A.R. (2020). A kinase-independent function for AURORA-A in replisome assembly during DNA replication initiation. Nucleic Acids Res..

[6] Chan C.S., Botstein D. (1993). Isolation and characterization of chromosome-gain and increase-in-ploidy mutants in yeast. Genetics.

[7] Lee E.C.Y., Frolov A., Li R., Ayala G., Greenberg N.M. (2006). Targeting Aurora kinases for the treatment of prostate cancer. Cancer Res..

[8] Du R., Huang C., Liu K., Li X., Dong Z. (2021). Targeting AURKA in Cancer: Molecular mechanisms and opportunities for Cancer therapy. Mol. Cancer.

[9] Furukawa T., Kanai N., Shiwaku H., Soga N., Uehara A., Horii A. (2006). AURKA is one of the downstream targets of MAPK1/ERK2 in pancreatic cancer. Oncogene.

[10] Cox D.G., Hankinson S.E., Hunter D.J. (2006). Polymorphisms of the AURKA (STK15/Aurora Kinase) gene and breast cancer risk (United States). Cancer Causes Control.

[11] Goos J.A., Coupe V.M., Diosdado B., Diemen D.-V., Karga C., Belien J.A., Carvalho B., van den Tol M.P., Verheul H.M., Geldof A.A. (2013). Aurora kinase A (AURKA) expression in colorectal cancer liver metastasis is associated with poor prognosis. Br. J. Cancer.

[12] Tang A., Gao K., Chu L., Zhang R., Yang J., Zheng J. (2017). Aurora kinases: Novel therapy targets in cancers. Oncotarget.

[13] Bavetsias V., Linardopoulos S. (2015). Aurora kinase inhibitors: Current status and outlook. Front. Oncol..

[14] Keen N., Taylor S. (2004). Aurora-kinase inhibitors as anticancer agents. Nat. Rev. Cancer.

[15] Borisa A.C., Bhatt H.G. (2017). A comprehensive review on Aurora kinase: Small molecule inhibitors and clinical trial studies. Eur. J. Med. Chem..

[16] Falchook G.S., Bastida C.C., Kurzrock R. (2015). Aurora kinase inhibitors in oncology clinical trials: Current state of the progress. Semin. Oncol..

[17] O’connor O.A., Ozcan M., Jacobsen E.D., Roncero J.M., Trotman J., Demeter J., Masszi T., Pereira J., Ramchandren R., Beaven A. (2019). Randomized phase III study of alisertib or investigator’s choice (selected single agent) in patients with relapsed or refractory peripheral T-cell lymphoma. J. Clin. Oncol..

[18] Chu Q.S.-C., Bouganim N., Fortier C., Zaknoen S., Stille J.R., Kremer J.D., Yuen E., Hui Y.-H., de la Peña A., Lithio A. (2021). Aurora kinase A inhibitor, LY3295668 erbumine: A phase 1 monotherapy safety study in patients with locally advanced or metastatic solid tumors. Investig. New Drugs.

[19] Rosenthal A., Kumar S., Hofmeister C., Laubach J., Vij R., Dueck A., Gano K., Stewart A.K. (2016). A Phase Ib Study of the combination of Aurora Kinase Inhibitor alisertib (MLN8237) and bortezomib in Relapsed or Refractory Multiple Myeloma. Br. J. Haematol..

[20] Macarulla T., Cervantes A., Elez E., Rodríguez-Braun E., Baselga J., Roselló S., Sala G., Blasco I., Danaee H., Lee Y. (2010). Phase I Study of the Selective Aurora A Kinase Inhibitor MLN8054 in Patients with Advanced Solid Tumors: Safety, Pharmacokinetics, and Pharmacodynamics MLN8054: Outcomes of a Phase I Study. Mol. Cancer Ther..

[21] Joukov V., De Nicolo A. (2018). Aurora-PLK1 cascades as key signaling modules in the regulation of mitosis. Sci. Signal..

[22] Gomez-Ferreria M.A., Rath U., Buster D.W., Chanda S.K., Caldwell J.S., Rines D.R., Sharp D.J. (2007). Human Cep192 is required for mitotic centrosome and spindle assembly. Curr. Biol..

[23] Joukov V., De Nicolo A., Rodriguez A., Walter J.C., Livingston D.M. (2010). Centrosomal protein of 192 kDa (Cep192) promotes centrosome-driven spindle assembly by engaging in organelle-specific Aurora A activation. Proc. Natl. Acad. Sci. USA.

[24] Joukov V., Walter J.C., De Nicolo A. (2014). The Cep192-organized aurora A-Plk1 cascade is essential for centrosome cycle and bipolar spindle assembly. Mol. Cell.

[25] Karthigeyan D., Prasad S.B.B., Shandilya J., Agrawal S., Kundu T.K. (2011). Biology of Aurora A kinase: Implications in cancer manifestation and therapy. Med. Res. Rev..

[26] Carmena M., Ruchaud S., Earnshaw W.C. (2009). Making the Auroras glow: Regulation of Aurora A and B kinase function by interacting proteins. Curr. Opin. Cell Biol..

[27] Hutterer A., Berdnik D., Wirtz-Peitz F., Žigman M., Schleiffer A., Knoblich J.A. (2006). Mitotic activation of the kinase Aurora-A requires its binding partner Bora. Dev. Cell.

[28] Zang J., Chen Y., Liu C., Lin S. (2022). Probing the Role of Aurora Kinase A Threonylation with Site-Specific Lysine Threonylation. ACS Chem. Biol..

[29] Byrne D.P., Shrestha S., Galler M., Cao M., Daly L.A., Campbell A.E., Eyers C.E., Veal E.A., Kannan N., Eyers P.A. (2020). Aurora A regulation by reversible cysteine oxidation reveals evolutionarily conserved redox control of Ser/Thr protein kinase activity. Sci. Signal..

[30] Lim D.C., Joukov V., Rettenmaier T.J., Kumagai A., Dunphy W.G., Wells J.A., Yaffe M.B. (2020). Redox priming promotes Aurora A activation during mitosis. Sci. Signal..

[31] Tsuchiya Y., Byrne D.P., Burgess S.G., Bormann J., Baković J., Huang Y., Zhyvoloup A., Yu B.Y.K., Peak-Chew S., Tran T. (2020). Covalent Aurora A regulation by the metabolic integrator coenzyme A. Redox Biol..

[32] Wang G.-F., Dong Q., Bai Y., Yuan J., Xu Q., Cao C., Liu X. (2017). Oxidative stress induces mitotic arrest by inhibiting Aurora A-involved mitotic spindle formation. Free. Radic. Biol. Med..

[33] Janeček M., Rossmann M., Sharma P., Emery A., Huggins D.J., Stockwell S.R., Stokes J.E., Tan Y.S., Almeida E.G., Hardwick B. (2016). Allosteric modulation of AURKA kinase activity by a small-molecule inhibitor of its protein-protein interaction with TPX2. Sci. Rep..

[34] McIntyre P.J., Collins P.M., Vrzal L.s., Birchall K., Arnold L.H., Mpamhanga C., Coombs P.J., Burgess S.G., Richards M.W., Winter A. (2017). Characterization of three druggable hot-spots in the Aurora-A/TPX2 interaction using biochemical, biophysical, and fragment-based approaches. ACS Chem. Biol..

[35] Asteriti I.A., Daidone F., Colotti G., Rinaldo S., Lavia P., Guarguaglini G., Paiardini A. (2017). Identification of small molecule inhibitors of the Aurora-A/TPX2 complex. Oncotarget.

[36] Cole D.J., Janecek M., Stokes J.E., Rossmann M., Faver J.C., McKenzie G.J., Venkitaraman A.R., Hyvönen M., Spring D.R., Huggins D.J. (2017). Computationally-guided optimization of small-molecule inhibitors of the Aurora A kinase–TPX2 protein–protein interaction. Chem. Commun..

[37] Bayliss R., Sardon T., Vernos I., Conti E. (2003). Structural basis of Aurora-A activation by TPX2 at the mitotic spindle. Mol. Cell.

[38] Kufer T.A., Silljé H.H., Körner R., Gruss O.J., Meraldi P., Nigg E.A. (2002). Human TPX2 is required for targeting Aurora-A kinase to the spindle. J. Cell Biol..

[39] Wittmann T., Wilm M., Karsenti E., Vernos I. (2000). TPX2, A novel xenopus MAP involved in spindle pole organization. J. Cell Biol..

[40] Zeng K., Bastos R.N., Barr F.A., Gruneberg U. (2010). Protein phosphatase 6 regulates mitotic spindle formation by controlling the T-loop phosphorylation state of Aurora A bound to its activator TPX2. J. Cell Biol..

[41] Dauch D., Rudalska R., Cossa G., Nault J.-C., Kang T.-W., Wuestefeld T., Hohmeyer A., Imbeaud S., Yevsa T., Hoenicke L. (2016). A MYC–aurora kinase A protein complex represents an actionable drug target in p53-altered liver cancer. Nat. Med..

[42] Eilers M., Eisenman R.N. (2008). Myc’s broad reach. Genes Dev..

[43] Meyer N., Penn L.Z. (2008). Reflecting on 25 years with MYC. Nat. Rev. Cancer.

[44] Richards M.W., Burgess S.G., Poon E., Carstensen A., Eilers M., Chesler L., Bayliss R. (2016). Structural basis of N-Myc binding by Aurora-A and its destabilization by kinase inhibitors. Proc. Natl. Acad. Sci. USA.

[45] Otto T., Horn S., Brockmann M., Eilers U., Schüttrumpf L., Popov N., Kenney A.M., Schulte J.H., Beijersbergen R., Christiansen H. (2009). Stabilization of N-Myc is a critical function of Aurora A in human neuroblastoma. Cancer Cell.

[46] Brockmann M., Poon E., Berry T., Carstensen A., Deubzer H.E., Rycak L., Jamin Y., Thway K., Robinson S.P., Roels F. (2013). Small molecule inhibitors of aurora-a induce proteasomal degradation of N-myc in childhood neuroblastoma. Cancer Cell.

[47] Faisal A., Vaughan L., Bavetsias V., Sun C., Atrash B., Avery S., Jamin Y., Robinson S.P., Workman P., Blagg J. (2011). The aurora kinase inhibitor CCT137690 downregulates MYCN and sensitizes MYCN-amplified neuroblastoma in vivo. Mol. Cancer Ther..

[48] Gustafson W.C., Meyerowitz J.G., Nekritz E.A., Chen J., Benes C., Charron E., Simonds E.F., Seeger R., Matthay K.K., Hertz N.T. (2014). Drugging MYCN through an allosteric transition in Aurora kinase A. Cancer Cell.

[49] Bellany F., Tsuchiya Y., Tran T.M., Chan A.E., Allan H., Gout I., Tabor A.B. (2020). Design and synthesis of Coenzyme A analogues as Aurora kinase A inhibitors: An exploration of the roles of the pyrophosphate and pantetheine moieties. Bioorganic Med. Chem..

[50] Dupré-Crochet S., Erard M., Nü O. (2013). ROS production in phagocytes: Why, when, and where?. J. Leukoc. Biol..

[51] Lambert A.J., Brand M.D. (2009). Reactive oxygen species production by mitochondria. Mitochondrial DNA.

[52] Del Río L.A., López-Huertas E. (2016). ROS generation in peroxisomes and its role in cell signaling. Plant Cell Physiol..

[53] Finkel T., Holbrook N.J. (2000). Oxidants, oxidative stress and the biology of ageing. Nature.

[54] Mittler R., Vanderauwera S., Suzuki N., Miller G., Tognetti V.B., Vandepoele K., Gollery M., Shulaev V., Van Breusegem F. (2011). ROS signaling: The new wave?. Trends Plant Sci..

[55] Zhang J., Wang X., Vikash V., Ye Q., Wu D., Liu Y., Dong W. (2016). ROS and ROS-mediated cellular signaling. Oxidative Med. Cell. Longev..

[56] da Veiga Moreira J., Peres S., Steyaert J.-M., Bigan E., Paulevé L., Nogueira M.L., Schwartz L. (2015). Cell cycle progression is regulated by intertwined redox oscillators. Theor. Biol. Med. Model..

[57] An B.C., Choi Y.-D., Oh I.-J., Kim J.H., Park J.-I., Lee S.-w. (2018). GPx3-mediated redox signaling arrests the cell cycle and acts as a tumor suppressor in lung cancer cell lines. PLoS ONE.

[58] Mailand N., Podtelejnikov A.V., Groth A., Mann M., Bartek J., Lukas J. (2002). Regulation of G2/M events by Cdc25A through phosphorylation-dependent modulation of its stability. EMBO J..

[59] Rudolph J. (2005). Redox regulation of the Cdc25 phosphatases. Antioxid. Redox Signal..

[60] Nilsson I., Hoffmann I. (2000). Cell cycle regulation by the Cdc25 phosphatase family. Prog. Cell Cycle Res..

[61] Boutros R., Dozier C., Ducommun B. (2006). The when and wheres of CDC25 phosphatases. Curr. Opin. Cell Biol..

[62] Savitsky P.A., Finkel T. (2002). Redox regulation of Cdc25C. J. Biol. Chem..

[63] Virshup D.M. (2000). Protein phosphatase 2A: A panoply of enzymes. Curr. Opin. Cell Biol..

[64] Hunt T. (2013). On the regulation of protein phosphatase 2A and its role in controlling entry into and exit from mitosis. Adv. Biol. Regul..

[65] Walter A.O., Seghezzi W., Korver W., Sheung J., Lees E. (2000). The mitotic serine/threonine kinase Aurora2/AIK is regulated by phosphorylation and degradation. Oncogene.

[66] Forester C.M., Maddox J., Louis J.V., Goris J., Virshup D.M. (2007). Control of mitotic exit by PP2A regulation of Cdc25C and Cdk1. Proc. Natl. Acad. Sci. USA.

[67] Margolis S.S., Perry J.A., Forester C.M., Nutt L.K., Guo Y., Jardim M.J., Thomenius M.J., Freel C.D., Darbandi R., Ahn J.-H. (2006). Role for the PP2A/B56δ phosphatase in regulating 14-3-3 release from Cdc25 to control mitosis. Cell.

[68] Low I.C.C., Loh T., Huang Y., Virshup D.M., Pervaiz S. (2014). Ser70 phosphorylation of Bcl-2 by selective tyrosine nitration of PP2A-B56δ stabilizes its antiapoptotic activity. Blood.

[69] Chen L., Liu L., Yin J., Luo Y., Huang S. (2009). Hydrogen peroxide-induced neuronal apoptosis is associated with inhibition of protein phosphatase 2A and 5, leading to activation of MAPK pathway. Int. J. Biochem. Cell Biol..

[70] Gu Y., Barzegar M., Chen X., Wu Y., Shang C., Mahdavian E., Salvatore B.A., Jiang S., Huang S. (2015). Fusarochromanone-induced reactive oxygen species results in activation of JNK cascade and cell death by inhibiting protein phosphatases 2A and 5. Oncotarget.

[71] Foley T.D., Petro L.A., Stredny C.M., Coppa T.M. (2007). Oxidative inhibition of protein phosphatase 2A activity: Role of catalytic subunit disulfides. Neurochem. Res..

[72] Sommer D., Coleman S., Swanson S.A., Stemmer P.M. (2002). Differential susceptibilities of serine/threonine phosphatases to oxidative and nitrosative stress. Arch. Biochem. Biophys..

[73] Janssens V., Longin S., Goris J. (2008). PP2A holoenzyme assembly: In cauda venenum (the sting is in the tail). Trends Biochem. Sci..

[74] Poole L.B., Nelson K.J. (2008). Discovering mechanisms of signaling-mediated cysteine oxidation. Curr. Opin. Chem. Biol..

[75] Reddie K.G., Carroll K.S. (2008). Expanding the functional diversity of proteins through cysteine oxidation. Curr. Opin. Chem. Biol..

[76] Bak D.W., Bechtel T.J., Falco J.A., Weerapana E. (2019). Cysteine reactivity across the subcellular universe. Curr. Opin. Chem. Biol..

[77] Netto L.E.S., de Oliveira M.A., Monteiro G., Demasi A.P.D., Cussiol J.R.R., Discola K.F., Demasi M., Silva G.M., Alves S.V., Faria V.G. (2007). Reactive cysteine in proteins: Protein folding, antioxidant defense, redox signaling and more. Comp. Biochem. Physiol. Part C: Toxicol. Pharmacol..

[78] Kallis G.-B., Holmgren A. (1980). Differential reactivity of the functional sulfhydryl groups of cysteine-32 and cysteine-35 present in the reduced form of thioredoxin from Escherichia coli. J. Biol. Chem..

[79] Dyson H.J., Tennant L.L., Holmgren A. (1991). Proton-transfer effects in the active-site region of Escherichia coli thioredoxin using two-dimensional proton NMR. Biochemistry.

[80] Nelson K.J., Day A.E., Zeng B.-B., King S.B., Poole L.B. (2008). Isotope-coded, iodoacetamide-based reagent to determine individual cysteine pKa values by matrix-assisted laser desorption/ionization time-of-flight mass spectrometry. Anal. Biochem..

[81] Sundaramoorthy E., Maiti S., Brahmachari S.K., Sengupta S. (2008). Predicting protein homocysteinylation targets based on dihedral strain energy and pKa of cysteines. Proteins: Struct. Funct. Bioinform..

[82] Madzelan P., Labunska T., Wilson M.A. (2012). Influence of peptide dipoles and hydrogen bonds on reactive cysteine pK a values in fission yeast DJ-1. FEBS J..

[83] Marino S.M., Gladyshev V.N. (2012). Analysis and functional prediction of reactive cysteine residues. J. Biol. Chem..

[84] Soylu İ., Marino S.M. (2016). Cy-preds: An algorithm and a web service for the analysis and prediction of cysteine reactivity. Proteins Struct. Funct. Bioinform..

[85] Hirota T., Kunitoku N., Sasayama T., Marumoto T., Zhang D., Nitta M., Hatakeyama K., Saya H. (2003). Aurora-A and an interacting activator, the LIM protein Ajuba, are required for mitotic commitment in human cells. Cell.

[86] Bai M., Ni J., Wu J., Wang B., Shen S., Yu L. (2014). A novel mechanism for activation of Aurora-A kinase by Ajuba. Gene.

[87] Cheetham G.M., Knegtel R.M., Coll J.T., Renwick S.B., Swenson L., Weber P., Lippke J.A., Austen D.A. (2002). Crystal structure of aurora-2, an oncogenic serine/threonine kinase. J. Biol. Chem..

[88] Nolen B., Taylor S., Ghosh G. (2004). Regulation of protein kinases: Controlling activity through activation segment conformation. Mol. Cell.

[89] Levinson N.M. (2018). The multifaceted allosteric regulation of Aurora kinase A. Biochem. J..

[90] Zorba A., Buosi V., Kutter S., Kern N., Pontiggia F., Cho Y.-J., Kern D. (2014). Molecular mechanism of Aurora A kinase autophosphorylation and its allosteric activation by TPX2. eLife.

[91] Eyers P.A., Erikson E., Chen L.G., Maller J.L. (2003). A novel mechanism for activation of the protein kinase Aurora A. Curr. Biol..

[92] Ruff E.F., Muretta J.M., Thompson A.R., Lake E.W., Cyphers S., Albanese S.K., Hanson S.M., Behr J.M., Thomas D.D., Chodera J.D. (2018). A dynamic mechanism for allosteric activation of Aurora kinase A by activation loop phosphorylation. eLife.

[93] Cyphers S., Ruff E.F., Behr J.M., Chodera J.D., Levinson N.M. (2017). A water-mediated allosteric network governs activation of Aurora kinase A. Nat. Chem. Biol..

[94] Bertolin G., Sizaire F., Herbomel G., Reboutier D., Prigent C., Tramier M. (2016). A FRET biosensor reveals spatiotemporal activation and functions of aurora kinase A in living cells. Nat. Commun..

[95] Lake E.W., Muretta J.M., Thompson A.R., Rasmussen D.M., Majumdar A., Faber E.B., Ruff E.F., Thomas D.D., Levinson N.M. (2018). Quantitative conformational profiling of kinase inhibitors reveals origins of selectivity for Aurora kinase activation states. Proc. Natl. Acad. Sci. USA.

[96] Dodson C.A., Bayliss R. (2012). Activation of Aurora-A kinase by protein partner binding and phosphorylation are independent and synergistic. J. Biol. Chem..

[97] Hammond D., Zeng K., Espert A., Bastos R.N., Baron R.D., Gruneberg U., Barr F.A. (2013). Melanoma-associated mutations in protein phosphatase 6 cause chromosome instability and DNA damage owing to dysregulated Aurora-A. J. Cell Sci..

[98] Toya M., Terasawa M., Nagata K., Iida Y., Sugimoto A. (2011). A kinase-independent role for Aurora A in the assembly of mitotic spindle microtubules in Caenorhabditis elegans embryos. Nat. Cell Biol..

[99] Dutertre S., Cazales M., Quaranta M., Froment C., Trabut V., Dozier C., Mirey G., Bouché J.-P., Theis-Febvre N., Schmitt E. (2004). Phosphorylation of CDC25B by Aurora-A at the centrosome contributes to the G2–M transition. J. Cell Sci..

[100] Giorgio M., Trinei M., Migliaccio E., Pelicci P.G. (2007). Hydrogen peroxide: A metabolic by-product or a common mediator of ageing signals?. Nat. Rev. Mol. Cell Biol..

[101] Veal E., Day A. (2011). Hydrogen peroxide as a signaling molecule. Antioxid. Redox Signal..

[102] Di Marzo N., Chisci E., Giovannoni R. (2018). The role of hydrogen peroxide in redox-dependent signaling: Homeostatic and pathological responses in mammalian cells. Cells.

[103] Lim J.M., Lee K.S., Woo H.A., Kang D., Rhee S.G. (2015). Control of the pericentrosomal H_2_O_2_ level by peroxiredoxin I is critical for mitotic progression. J. Cell Biol..

[104] Rhee S.G. (2006). H_2_O_2_, a necessary evil for cell signaling. Science.

[105] Kwon J., Lee S.-R., Yang K.-S., Ahn Y., Kim Y.J., Stadtman E.R., Rhee S.G. (2004). Reversible oxidation and inactivation of the tumor suppressor PTEN in cells stimulated with peptide growth factors. Proc. Natl. Acad. Sci. USA.

[106] Deshpande N.N., Sorescu D., Seshiah P., Ushio-Fukai M., Akers M., Yin Q., Griendling K.K. (2002). Mechanism of hydrogen peroxide-induced cell cycle arrest in vascular smooth muscle. Antioxid. Redox Signal..

[107] Havens C.G., Ho A., Yoshioka N., Dowdy S.F. (2006). Regulation of late G1/S phase transition and APCCdh1 by reactive oxygen species. Mol. Cell. Biol..

[108] Goswami P.C., Sheren J., Albee L.D., Parsian A., Sim J.E., Ridnour L.A., Higashikubo R., Gius D., Hunt C.R., Spitz D.R. (2000). Cell cycle-coupled variation in topoisomerase IIα mRNA is regulated by the 3′-untranslated region: Possible role of redox-sensitive protein binding in mRNA accumulation. J. Biol. Chem..

[109] Yamaura M., Mitsushita J., Furuta S., Kiniwa Y., Ashida A., Goto Y., Shang W.H., Kubodera M., Kato M., Takata M. (2009). NADPH oxidase 4 contributes to transformation phenotype of melanoma cells by regulating G2-M cell cycle progression. Cancer Res..

[110] Hochegger H., Hégarat N., Pereira-Leal J. (2013). Aurora at the pole and equator: Overlapping functions of Aurora kinases in the mitotic spindle. Open Biol..

[111] Marumoto T., Zhang D., Saya H. (2005). Aurora-A—A guardian of poles. Nat. Rev. Cancer.

[112] Gout I. (2018). Coenzyme A, protein CoAlation and redox regulation in mammalian cells. Biochem. Soc. Trans..

[113] Leonardi R., Zhang Y.-M., Rock C.O., Jackowski S. (2005). Coenzyme A: Back in action. Prog. Lipid Res..

[114] Baković J., Yu B.Y.K., Silva D., Chew S.P., Kim S., Ahn S.-H., Palmer L., Aloum L., Stanzani G., Malanchuk O. (2019). A key metabolic integrator, coenzyme A, modulates the activity of peroxiredoxin 5 via covalent modification. Mol. Cell. Biochem..

[115] Tsuchiya Y., Zhyvoloup A., Baković J., Thomas N., Yu B.Y.K., Das S., Orengo C., Newell C., Ward J., Saladino G. (2018). Protein CoAlation and antioxidant function of coenzyme A in prokaryotic cells. Biochem. J..

[116] McCoy F., Darbandi R., Lee H.C., Bharatham K., Moldoveanu T., Grace C.R., Dodd K., Lin W., Chen S.-I., Tangallapally R.P. (2013). Metabolic activation of CaMKII by coenzyme A. Mol. Cell.

[117] Ford D.A., Horner C.C., Gross R.W. (1998). Protein kinase C acylation by palmitoyl coenzyme A facilitates its translocation to membranes. Biochemistry.

[118] Guimarães C.R., Rai B.K., Munchhof M.J., Liu S., Wang J., Bhattacharya S.K., Buckbinder L. (2011). Understanding the impact of the P-loop conformation on kinase selectivity. J. Chem. Inf. Model..

[119] La Sala G., Riccardi L., Gaspari R., Cavalli A., Hantschel O., De Vivo M. (2016). HRD motif as the central hub of the signaling network for activation loop autophosphorylation in Abl kinase. J. Chem. Theory Comput..

[120] Abdeldayem A., Raouf Y.S., Constantinescu S.N., Moriggl R., Gunning P.T. (2020). Advances in covalent kinase inhibitors. Chem. Soc. Rev..

